# ﻿On the genus *Coccophagus* Westwood (Hymenoptera, Aphelinidae) from Xishuangbanna Rainforest. Contribution I: Two new species of the *Coccophagusvarius* group, with an identification key and phylogenetic analysis

**DOI:** 10.3897/zookeys.1091.80065

**Published:** 2022-04-01

**Authors:** Yao-guang Qin, Hai-feng Chen, Cheng-de Li, Ye Chen

**Affiliations:** 1 Hebei Key Laboratory of Animal Diversity, College of Life Science, Langfang Normal University, Langfang, 065000, China Langfang Normal University Langfang China; 2 School of Forestry, Northeast Forestry University, Harbin, 150040, China Northeast Forestry University Harbin China

**Keywords:** Chalcidoidea, Coccophaginae, parasitoid wasp, taxonomy

## Abstract

Two new species belonging to the *varius* group of *Coccophagus*, *C.breviclavulus***sp. nov.** and *C.perlucidus***sp. nov.**, are described from Xishuangbanna Rainforest (China, Yunnan). *Coccophagusanchoroides* (Huang) and *C.yunnana* Wang, Huang & Polaszek are recorded. A tentative key to world species of this group is provided. Partial nuclear ribosomal 28S-D2 of these four species and other six species were sequenced and subjected to a phylogenetic analysis. Phylogeny of *C.varius* group is discussed.

## ﻿Introduction

In 2019 and 2020, we undertook extensive sampling and surveying of arthropods in the canopy of the Xishuangbanna Rainforest, and collected some specimens belonging to the genus *Coccophagus* Westwood. In the present paper, some of the specimens within the *Coccophagusvarius* group are studied, as the first contribution to the genus *Coccophagus* from Xishuangbanna Rainforest (Yunnan Province).

*Coccophagus* Westwood, 1833 is the second largest genus of Aphelinidae,and currently contains 271 valid species, of which 36 species are known from China (Chen and Li 2017; Noyes 2019; Wang et al. 2020). The females of *Coccophagus* are endoparasitoids of scale insects (Hemiptera: Coccomorpha), mainly of soft scales (Coccidae) and rarely of mealybugs (Pseudococcidae); males are generally hyperparasitoids on other primary parasitoids, including conspecific females (Clausen 1978; Hayat 1988). Currently, *Coccophagus* is divided into three subgenera: *Dicoccophagus*Sugonjaev (1994), *Polycoccophagus*Sugonjaev (1976) and *Coccophaguss. str.* (Chen and Li 2017). According to Compere (1931), Annecke and Insley (1974), Hayat (1988, 1992, 1998) and Myartseva and Ruíz-Cancino (2005), eight species groups have been recognized under *Coccophagus s. str.: lycimnia*, *ochraceus*, *malthusi*, *pseudococci*, *varius*, *zebratus*, *tschirchii*, and *redini* groups.

The *Coccophagusvarius* species group was proposed by Hayat (1988) for those species which were previously placed in the genus *Prococcophagus* Silvestri. The status of *Prococcophagus* was first queried by Hayat (1983), and he stated *Prococcophagus* did not merit a separate status and may ultimately be treated as a species group of *Coccophagus*. Later, Shafee et al. (1985) and Viggiani (1985) both supported the synonymy of *Prococcophagus* under *Coccophagus*. The *varius* group of *Coccophagus* can be recognised by the following combination of characters: scape flattened and expanded ventrally and less than 3.0× as long as wide (with some exceptions: not flattened and expanded, e.g. *Coccophagusperlucidus*, cf. Fig. 19); body with contrasting brown and silver-white areas; antennomeres with white and dark segments; fore wing with distinct infuscation (with some exceptions: fore wing uniformly hyaline), the infuscate area with dark brown setae, and the hyaline area with transparent setae. Apart from the above characters noted by most authors (Hayat 1988, 1998; Myartseva 2004; Wang et al. 2020), our specimens (n=19) have two small patches posterior to each posterior ocellus respectively. Wang et al (2020) conducted phylogenetic analysis to discuss the systematic status of this species group based on 28S-D2 rDNA sequences though only including two species of this species group.

Until the present study, *C.varius* species group included 24 species which were originally found in Palaearctic (1 species), Oriental (12), Australian (4), Ethiopian (4) and Neotropical Regions (3) (Noyes 2019; Wang et al. 2020). Herein, two new species from Xishuangbanna Rainforest (Yunnan, China) are added to the Oriental region, *C.anchoroides* is newly reported from Yunnan Province, 28S-D2 rDNA of *C.yunnana* is sequenced for the first time and a key to all the known species in this group is provided. In addition, phylogenetic analyses including 12 online 28S-D2 rDNA sequences together with our *de novo* data, which represented five species groups of *Coccophagus*, were carried out to assess the systematic status.

## ﻿Materials and methods

### ﻿Morphological study

Samples were obtained using a pyrethroid fog generated from a thermal fogger (Swingfog SN50, Germany, Model 2610E, Series 3). Specimens were dissected and mounted in Canada Balsam on slides, following the method described by Noyes (1982). Specimens in ethanol and on slides were photographed and then the images were processed, following Chen and Chen (2021). Scale bars are 100 μm except where otherwise indicated. All specimens listed below are deposited in Langfang Normal University, Langfang, China.

Terminology follows the Hymenoptera Anatomy Consortium (2021). The following abbreviations are used in the text: C1–3, clavomeres 1–3; F1–3, funicle segments 1–3; Gt_1_, Gt_2_ etc., tergites 1, 2, etc. of gaster.

### ﻿Abbreviations for depositories as follows

**FAFU** Fujian Agriculture and Forestry University, Fuzhou 350002, China;

**LFNU** Langfang Normal University, 065000, China.

### ﻿DNA extraction, amplification, and sequencing

Genomic DNA extraction was from the entire body of female adults. The body was destroyed and performed using the DNeasy Blood & Tissue Kit (Qiagen GmbH, Hilden, Germany) following the manufacturer’s protocols. The forward and reverse primers used for amplifying the D2 region of 28S rDNA gene were [F] 5’-CGT GTT GCT TGA TAG TGC AGC-3’and [R] 5’-TTG GTC CGT GTT TCA AGA CGG G-3’respectively (Campbell et al. 1994). The amplification program was: initial denaturation step at 95 °C for 5min, denaturation step at 95 °C for 30s, annealing for 45s at 58 °C, and extension at 72 °C for 1min, with 40 cycles being performed, and final extension at 72 °C for 5min. Each PCR product was subjected to electrophoresis on 1% agarose gel, and positive products were sequenced directly in both directions using BigDye v3.1 on an ABI 3730xl DNA Analyser (Applied Biosystems). Generated sequences were deposited in GenBank (accession numbers: OM095389–OM095398).

### ﻿Phylogenetic analysis

To investigate the phylogenetic relationship between the *Coccophagusvarius* group and other *Coccophagus* species, Bayesian inference (BI) and Maximum likelihood (ML) were used to reconstruct phylogenetic trees using 28S-D2 rDNA dataset. The dataset included 22 ingroups (12 online data and 10 produced data in this study), representing 22 species and 5 species groups of *Coccophagus*, and two 28S online sequences of the genus *Coccobius* were chosen as outgroups. The details of taxa are shown in Table 1. The 28S-D2 rDNA sequences were aligned with MAFFT (Katoh et al. 2002) using the Q-INS-i algorithm (Katoh and Toh 2008). BI tree was obtained with MrBayes 3.2 (Ronquist et al. 2012). The best-fit model SYM+I+G for BI analysis was estimated using jModelTest v2.1.3 (Darriba et al. 2012) and selected based on the Bayesian Information Criterion (BIC) (Luo et al. 2010). To ensure that the average standard deviation of split frequencies was less than 0.01, 10 million generations were run with sampling every 1000 generations. Node support was assessed by posterior probability (PP). ML tree was inferred using IQ-TREE, version 1.6 (Nguyen et al. 2015), with the best-fit model automatically selected by ModelFinder (Kalyaanamoorthy et al. 2017). Branch support (BS) was estimated using ultrafast bootstrap with 1000 replicates (Hoang et al. 2018).

**Table 1.** 28S-D2 rDNA of *Coccophagus* and outgroups used in this study.

Species	Group	GenBank Accession No.	Reference
S1 *C.yunnana*	*varius*	OM095389	This study
S2 *C.breviclavulus*	*varius*	OM095390	This study
S3 *C.longifasciatus*	*ochraceus*	OM095391	This study
S4 *C.chloropulvinariae*	*malthusi*	OM095392	This study
S5 *C.candidus*	*malthusi*	OM095393	This study
S6 *C.* sp.	*lycimnia*	OM095394	This study
S7 *C.* sp.	*pseudococci*	OM095395	This study
S8 *C.* sp.	*lycimnia*	OM095396	This study
S9 *C.perlucidus*	*varius*	OM095397	This study
S10 *C.anchoroides*	*varius*	OM095398	This study
*C.fumadus*	*varius*	MT677530.1	Wang et al. 2020
*C.bivittatus*	*ochraceus*	KY605784.1	Zhou et al. 2017
*C.ceroplastae*	*lycimnia*	KY605741.1	Zhou et al. 2017
*C.yoshidae*	*malthusi*	MH455871.1	Amouroux et al. 2019
*C.lycimnia*	*lycimnia*	KY605608.1	Zhou et al. 2017
*C.cowperi*	*lycimnia*	HM856875.1	Rugman-Jones et al. 2011
*C.semicircularis*	*malthusi*	KY605779.1	Zhou et al. 2017
*C.scutellaris*	*malthusi*	JN623562.1	Munro et al. 2011
*C.ishiii*	*malthusi*	KY605777.1	Zhou et al. 2017
*C.nigricorpus*	*malthusi*	KY605646.1	Zhou et al. 2017
*C.bogoriensis*	*lycimnia*	KY605553.1	Zhou et al. 2017
*C.japonicus*	*lycimnia*	KY605542.1	Zhou et al. 2017
*Coccobius* sp. D1492	Outgroup	AY599373.1	Gillespie et al. 2005
*Coccobius* sp. D1387	Outgroup	AY599372.1	Gillespie et al. 2005

## ﻿Results

### ﻿Key to species of *Coccophagusvarius* group (females)

**Table:** 

1	Scape normal, not expanded ventrally, at least 3.0× as long as wide	**2**
–	Scape flattened and expanded ventrally, less than 3.0× as long as wide	**5**
2(1)	Scape white, with a narrow brown stripe along the middle, and 3.0× as long as wide; funicle white dorsally and dark ventrally	***C.asterolecanii* (Dozier, 1932)**
–	Scape entirely white, or mostly white, with two short dark stripes, or white dorsally and dark ventrally, and more than 3.0× as long as wide; funicle entirely white or only with F1 dark basally	**3**
3(2)	Scape and pedicel more or less with dark areas	**4**
–	Scape and pedicel white	***C.lii* (Huang, 1994)**
4(3)	Scape white but with two short dark stripes apically; mid lobe of mesoscutum yellow or brown-yellow, with small dark areas posteriorly; mesoscutellum with two brown patches posteriorly (cf. fig. 85C in Huang 1994,); gaster largely yellow, with six dark brown cross bands; ovipositor shorter than mesotibia (0.91×)	***C.albifuniculatus* (Huang, 1994)**
–	Scape white dorsally and mostly dark ventrally as in Fig. 19; mid lobe of mesoscutum with a large dark patch anteriorly; mesoscutellum orange except brown posteriorly, and with a small brown inverted triangle anteriorly (Fig. 20); gaster with white and dark tergites as in Fig. 24, ovipositor 1.2× as long as wide	***C.perlucidus* Chen & Li, sp. nov.**
5(1)	G_t7_ elongate and pointed at apex, appearing like a caudate process (cf. Huang 1994, fig. 87C), ovipositor 2.24× as long as mesotibia, third valvula 3.0× as long as mesobasitarsus	***C.caudatus* (Huang, 1994)**
–	G_t7_ not elongate, ovipositor less than 2.0× mesotibia in length, third valvula clearly less than 3.0× mesobasitarsus in length	**6**
6(5)	Fore wing uniformly hyaline, without infuscated area (cf. fig. 91B in Huang 1994)	**7**
–	Fore wing with infuscate area	**9**
7(6)	Flagellomeres more paler; except F1 basally, C1 and C2 dark	***C.equifuniculatus* (Huang, 1994)**
–	Flagellomeres black	**8**
8(7)	Scape black, abdomen (as Compere 1936 noted) black and with a yellow band at base; ovipositor apparently not exserted	***C.nympha* (Girault, 1915)**
–	Scape black but with base and apex yellow, abdomen black without yellow markings; ovipositor exserted	***C.argentiscutellum* (Girault, 1915)**
9(6)	Fore wing with a conspicuous arched hyaline band at preapical area and infuscated apically (cf. fig. 6 in Compere 1936)	***C.aurantifrons* (Compere, 1936)**
–	Forewing hyaline apically (Figs 5, 13, 29) and without that hyaline band	10
10(9)	Fore wing with a basal hyaline area extending outward one half the length of the blade on the posterior part (as noted by Compere 1936)	***C.hispaniolae* (Dozier, 1932)**
–	Otherwise	**11**
11(10)	Pedicel and F1 pale	***C.mixtus* (Girault, 1915)**
–	Pedicel with pale and dark areas; F1 completely dark or with pale and dark areas	**12**
12(11)	All flagellomeres dark or mostly dark only with F2, F3 and C1 having small pale areas dorsally	**13**
–	Flagellomeres at least with one segment completely pale	**14**
13(12)	Scape largely white on outer surface, black on both dorsal and ventral margins; axillae yellow, with fuscous median spot; legs white	***C.tobiasi* Myartseva, 2004**
–	Scape (cf. fig. 6 in Annecke and Mynhardt 1979) largely brownish black on outer surface, pale dorsally and with a white curving lateral band; axillae dark brown; legs white with extensive dark markings	***C.neserorum* (Annecke & Mynhardt, 1979)**
14(12)	F2 completely pale	**15**
–	F2 with dark area	**20**
15(14)	Mid lobe of mesoscutum with a dark anchor shaped patch (Figs 1, 25)	**16**
–	Mid lobe of mesoscutum generally yellowish brown or brown, with dark or pale streaks	**17**
16(15)	Scape with dorsal margin and a median band white (cf. Fig. 3), F1 largely dark brown, C3 pale; gaster with 4 dark brown bands on Gt_1_–Gt_4_. F1 slightly longer than wide, F2 1.2× as long as wide	***C.anchoroides* (Huang, 1994)**
–	Scape with a hook-like white streak medially except the white dorsal margin (Fig. 27), F1 pale, C3 dark; gaster with 6 dark brown bands on Gt_1_–Gt_6_ (Fig. 32). F1 1.3–1.5× as long as wide, F2 1.6–1.7× as long as wide	***C.yunnana* Wang, Huang & Polaszek, 2020**
17(15)	Scape largely pale, with two dark broad streaks distally (Fig. 11)	***C.breviclavulus* Chen & Li, sp. nov.**
–	Scape differently coloured, with more extensive dark area	**18**
18(17)	Scape extremely expanded, 1.87× as long as wide; mesoscutellum yellowish brown, with anterior margin and two dark patches (cf. fig. 89C in Huang 1994) on posterior half	***C.dilatatus* (Huang, 1994)**
–	Scape more than 2.0× as long as wide; mesoscutellum without patches on posterior area	**9**
19(18)	Mid lobe of mesoscutum yellowish brown, F3 completely white; pedicel subequal to F1 in length; mesotibial spur slightly longer than corresponding basitarsus	***C.pellucidus* (Huang, 1994)**
–	Mid lobe of mesoscutum orange brown to brown, with a brown median longitudinal streak, F3 with a brown irregular patch distally; pedicel obviously longer than F1 (cf. fig. 292 in Hayat 1998); mesotibial spur slightly shorter than corresponding basitarsus	***C.zeyai* Hayat, 1998**
20(14)	F3 dark	**21**
–	F3 pale	**23**
21(20)	Fore wing with a hyaline area bearing transparent setae below basal half of marginal vein (cf. fig. 300 in Hayat 1998)	***C.narendrani* Hayat & Zeya, 1993**
–	Fore wing infuscated below marginal vein	**22**
22(21)	Scape brown, and with dorsal margin and a median streak white (cf. fig. 71 in Hayat and Khan 2010); pronotum silvery white, mesally dark; mesoscutellum brown	***C.fumadus* Hayat, 2010**
–	Scape white, and with dark margins; pronotum with collar brown, rest part sordid white; mesoscutellum reddish orange, and with two brown patches	***C.nipponicus* (Ishihara, 1977)**
23(20)	F1 dark ventrally and fading to white above; pedicel slender, 2.0× as long as wide, obviously longer than F1 (cf. fig. 10 in Annecke and Mynhardt 1979)	***C.eusaissetiae* Özdikmen, 2011**
–	F1 completely dark; pedicel less than 2.0× as long as wide, at most slightly longer than F1	**24**
24(23)	F1 with ventral margin shortest among funicle segments, without sensillum (cf. fig. 2 in Hayat 1988)…	***C.srilankensis* Hayat, 1988**
–	F1 with ventral margin longest among funicle segments, with sensilla (cf. fig. 5 in Silvestri 1915 and figs 1, 13 in Annecke and Mynhardt 1979)	**25**
25(24)	Scape (cf. fig. 13 in Annecke and Mynhardt 1979) with two largely separated, dark streaks on outer surface; pedicel whitish with only ventral edge rather narrowly brown or blackish brown	***C.probus* (Annecke & Mynhardt, 1979)**
–	Scape (cf. fig. 1 in Annecke and Mynhardt 1979) with extensively streak on outer surface, the dark streaks merging apically and basally; pedicel with ventral one half black, remainder pale	***C.varius* (Silvestri, 1915)**

#### ﻿ Coccophagus anchoroides

Taxon classificationAnimaliaHymenopteraAphelinidae

(Huang)

9E94E645-AA38-59E7-A071-CC42F942AB89

Figs 1–8

Prococcophagus anchoroides Huang, 1994: 259. Holotype ♀, China, FAFU, not examined.
Coccophagus anchoroides (Huang): Xu & Huang, 2004: 362; Wang et al. 2020: 1883.

##### Material examined.

1♀ [on slide, C202007-1]; Yunnan Province; Xishuangbanna; Mengla County; Menglun Town; 21°54.24'N, 101°16'E; 541m a.s.l.; 13 May 2019; Z.-l. Bai, Z.-g. Chen, C. Wang, H. Yu leg.; LFNU. 1♀ [on slide, C202009-2]; Yunnan Province; Xishuangbanna; Mengla County; Menglun Town; 21°54.33'N, 101°16.78'E; 616m a.s.l.; 26 Apr. 2019; Z.-l. Bai, Z.-g. Chen, C. Wang, Y.-f. Tong, H. Yu leg.; LFNU. 1♀ [destroyed for DNA extraction]; Yunnan Province; Xishuangbanna; Mengla County; Menglun Town; 21°54.18'N, 101°16.71'E; 606m a.s.l.; 5 May. 2019; Z.-l. Bai, Z.-g. Chen, C. Wang, Y.-f. Tong, H. Yu leg.

**Figures 1–8.**
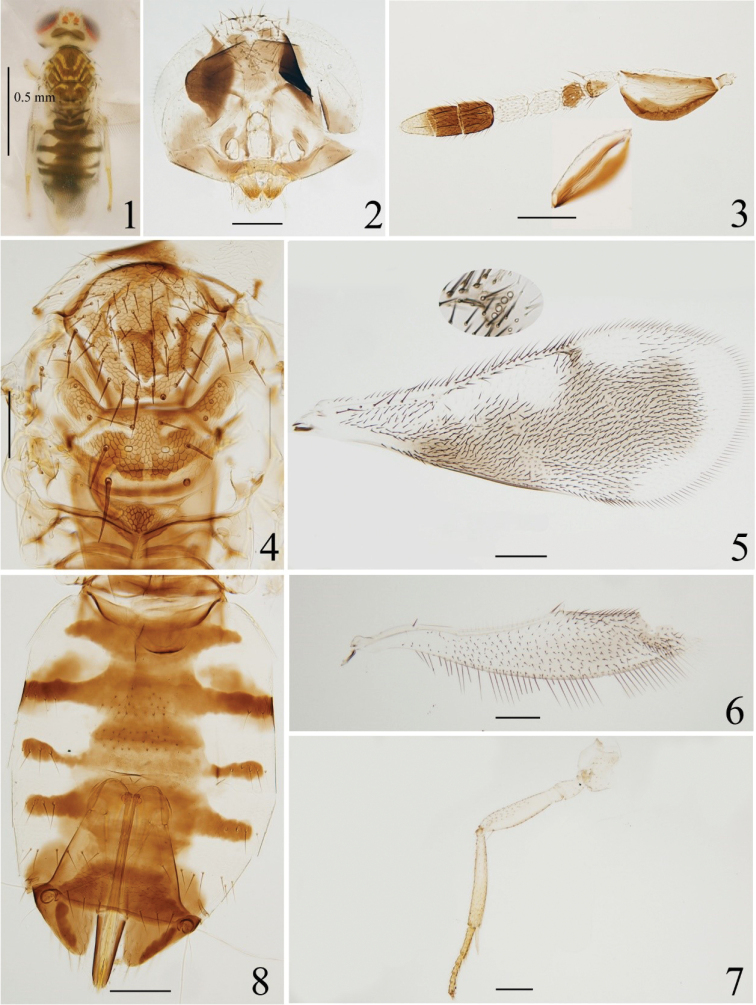
*Coccophagusanchoroides***1** body, dorsal view **2** head **3** antenna, inset shows the colour of outer surface of scape **4** mesosoma **5** fore wing **6** hind wing **7** mid leg **8** metasoma.

Professor Jian Huang (FAFU) confirmed our identification. Our specimens agree well with the original description in Huang (1994). A minor difference should be noted: mesoscutellum (Figs 1, 4) of our specimens with two yellow curved stripes anteriorly like *C.yunnana*, but in the original description mesoscutellum without yellow markings anteriorly (cf. fig. 90C in Huang 1994). Here we provided the digital images and DNA sequence for references.

##### Host.

Unknown.

##### Distribution.

China (Xishuangbanna of Yunnan Province [new record], Fujian).

#### ﻿ Coccophagus breviclavulus

Taxon classificationAnimaliaHymenopteraAphelinidae

Chen & Li sp. nov.

9535D38C-9531-5FBB-8A53-8E307D2AE8B5

http://zoobank.org/AC0A2216-A462-4121-88FB-A3EE97B4FBDF

Figs 9–16

##### Type material.

***Holotype***: China • ♀; Yunnan Province; Xishuangbanna; Mengla County; Menglun Town; 21°53.89'N, 101°16.72'E; 568 m a.s.l.; 22 May. 2019; Z.-l. Bai, Z.-g. Chen, C. Wang, H. Yu leg.; LFNU C202108-1 [on slide]. ***Paratypes***: 4♀♀ [3♀♀ on slides, C202108-2–C202108-4; 1♀ destroyed for DNA extraction]; same data as holotype; LFNU.

##### Diagnosis.

**Figures 9–16.**
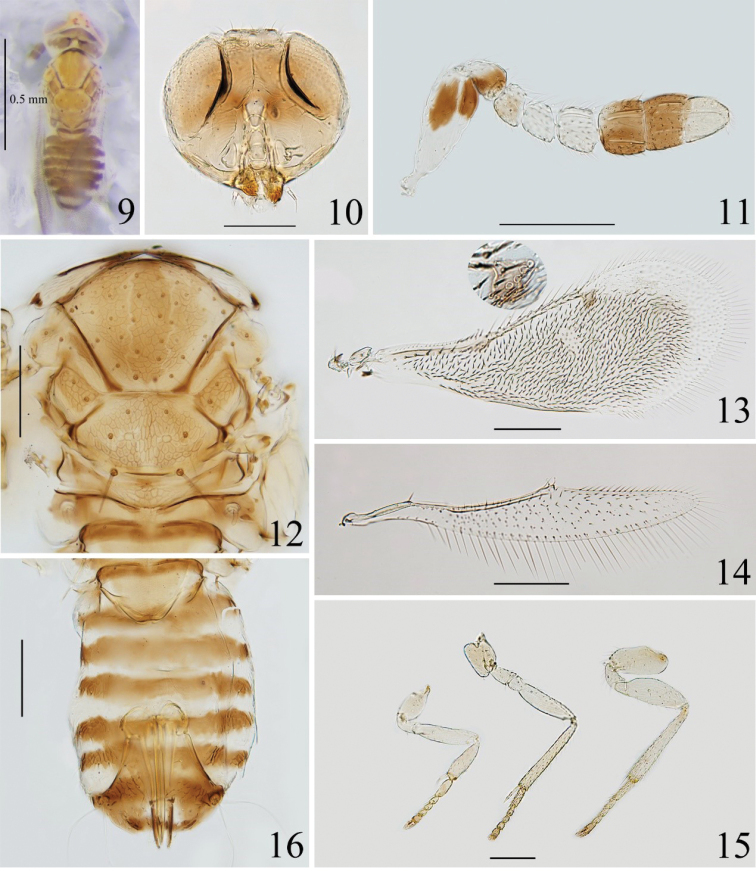
*Coccophagusbreviclavulus* sp. nov. **9** body, dorsal view **10** head **11** antenna **12** mesosoma **13** fore wing **14** hind wing **15** legs, from left to right: fore-, mid- and hind-leg **16** metasoma.

*Coccophagusbreviclavulus* sp. nov. can be distinguished from females of other species in this genus by the following combination of characters: scape largely white, and with two broad and short dark streaks distally (Fig. 3); F2 and F3 white; mesosoma (Fig. 12) most brown, with two longitudinal yellow lines medially on mid lobe of mesoscutum; metasoma largely dark brown as in Figs 9, 16; F1–F3 with the same length; clavomeres obviously wider than funicle segments.

##### Description.

**Female.** Length 0.7–0.9 mm; holotype 0.9 mm.

***Colour*.** Head (Fig. 10), in frontal view, mostly white; in dorsal view, vertex yellow, ocelli red-brown, eyes pale red and with two small dark patches behind each posterior ocellus (Fig. 9). Occiput brown above foramen, and with two dark brown suboval patches lateral to foramen, the remaining parts of occiput white. Scape (Fig. 11) with a dark broad streak on distal half of outer surface and on apex of ventral surface each, remainder parts white; pedicel dark brown except dorsal margin white; F1 suffused with brown, F2 and F3 white, C1 and C2 dark brown, C3 yellowish white. Mandible brown. Pronotum dark medially and white laterally; mid lobe of mesoscutum (Fig. 12) mostly brown, with two longitudinal yellow line medially, lateral and posterior edges yellow; side lobe of mesoscutum largely yellow, with a brown patch anteriorly, and with interior edge dark; notaulus dark; axilla dark brown, with lateral edge yellow; mesoscutellum brown except yellow margins; metanotum brown; propodeum brown with anterior and posterior margins and lateral sides dark brown. Fore wing (Fig. 13) largely infuscated and hyaline apically, with stigma vein brown; hind wing (Fig. 14) hyaline. Legs (Fig. 15) pale, with last tarsi brown. Metasoma (Fig. 9) with petiole dark brown on anterior half part and yellow posteriorly; Gt_1_–Gt_5_ largely dark brown and yellow on posterior margin of each tergite, Gt_6_ and Gt_7_ dark brown. Ovipositor with outer plates and third valvula dark brown. Ventral part of body generally pale.

***Head*** (Fig. 10), in frontal view, 0.8–0.9× as high as wide. Ocellar triangle with apical angle almost right-angled. Mandible tridentate. Antenna (Fig. 11) with scape 2.0–2.6× as long as wide; pedicel 1.2–1.4× as long as wide, 1.4× length of F1; F1–F3 ventrally connected, F1 with ventral length 1.7× dorsal length, and as long as wide; F2 about same size as F1; F3 0.9–1.0× as long as wide, as long as but a little wider than F1 and F2; clava with the second septum oblique, 1.2–1.4× length of funicle, and obviously wider than funicle segment. F1 without longitudinal sensilla, other flagellomeres with the following number of longitudinal sensilla successively: 1, 2, 2, 2, 2.

***Mesosoma*** (Fig. 12). Dorsum of mesosoma finely reticulate. Mid lobe of mesoscutum with approximately 40 setae, 0.8× as long as wide, 1.5× length of mesoscutellum; each side lobe of the mesoscutum with 3 setae; each axilla with 3 setae; mesoscutellum 0.6× as long as wide, with 3 pairs of setae. Distance between anterior pair of scutellar setae 0.5× and 0.6× that between median and posterior pair respectively. Placoid sensilla mesad of the median scutellar setae, and the distance between placoid sensilla about equal to that of anterior scutellar setae. Metanotum slightly longer than propodeum.

***Wings*.** Fore wing (Fig. 13) 2.5–2.8× as long as wide, marginal setae long and 0.15× wing width. Costal cell 0.8–0.9× length of marginal vein, bearing 1 row of setae and with the distal 6 setae long and coarse; submarginal vein with 6 setae; marginal vein with 9 long setae along anterior margin; postmarginal vein absent; stigmal vein (Fig. 13, inset) swollen posteriorly and with sensilla arranged in 1 line. Hind wing (Fig. 14) 5.5–6.6× as long as wide, with marginal setae 0.7–0.8× wing width.

***Legs*** (Fig. 15). Mesotibial spur as long as corresponding basitarsus.

***Metasoma*** (Fig. 16). Lateral sides of gaster, Gt_6_, posterior of Gt_7_ clearly reticulated. Setation of tergites on dorsal surface as followings: Gt_2_ with 2 and 3 setae (short for 2+3) on left and right side respectively, Gt_3_ 2 or 3+2, Gt_4_ 3+3, Gt_5_ 4+4 or 5+5, Gt_6_ with 6 arranged in a line, Gt_7_ with 8 setae arranged in two lines. Ovipositor originating from apex of Gt_3_, 1.0–1.1× as long as mesotibia, and slightly exerted. Gt_7_ 0.3× as long as wide. Second valvifer 1.9–2.0× as long as third valvula; the latter 1.1–1.3× as long as mesobasitarsus.

**Male.** Unknown.

##### Host.

Unknown.

##### Etymology.

The specific name refers to the scape with short streaks distally.

##### Distribution.

China (Xishuangbanna of Yunnan Province).

##### Comments.

This new species is similar to *C.anchoroides* but can be distinguished from the latter by the following characters: (1) scape largely white, and with two dark broad streaks distally (vs largely dark, with dorsal margin and a median streak white, cf. Fig. 5 and fig. 90A in Huang 1994); (2) mesoscutum without the anchor shaped patch (vs with, cf. Figs 1, 4); (3) Gt_1_–Gt_5_ with 5 brown transverse band (vs 4, cf. Fig. 8); (4) funicle segments equal in length, and each segment as long as wide (vs F1 shortest, F2 and F3 longer than wide); (5) fore wing with dark setae and without narrow hyaline area posterior to marginal vein (vs with a narrow hyaline area bearing fine pale setae, cf. Fig. 5); (6) ovipositor 1.0–1.1× as long as mesotibia (vs 1.3–1.6×).

#### ﻿ Coccophagus perlucidus

Taxon classificationAnimaliaHymenopteraAphelinidae

Chen & Li sp. nov.

BD2DFDAE-ECAC-58F2-A85A-2315842AB445

http://zoobank.org/2AFFB554-C935-4079-BACD-977C8D598DE6

Figs 17–24

##### Type material.

***Holotype***: China • ♀ [on slide, C202108-9]; Yunnan Province; Xishuangbanna; Mengla County; Menglun Town; 21°53.59'N, 101°17.29'E; 546 m a.s.l.; 4 May. 2019; Z.-l. Bai, Z.-g. Chen, C. Wang, Y.-f. Tong, H. Yu leg.; LFNU. ***Paratypes***: 1♀ [on slide, C202012-1]; Yunnan Province; Xishuangbanna; Mengla County; Menglun Town; 21°54.33'N, 101°16.78'E; 616m a.s.l.; 26 Apr. 2019; Z.-l. Bai, Z.-g. Chen, C. Wang, Y.-f. Tong, H. Yu leg.; LFNU. 1♀ [destroyed for DNA extraction]; Yunnan Province; Xishuangbanna; Mengla County; Menglun Town; 21°54.18'N, 101°16.71'E; 606 m a.s.l.; 5 May. 2019; Z.-l. Bai, Z.-g. Chen, C. Wang, Y.-f. Tong, H. Yu leg.; LFNU.

**Figures 17–24.**
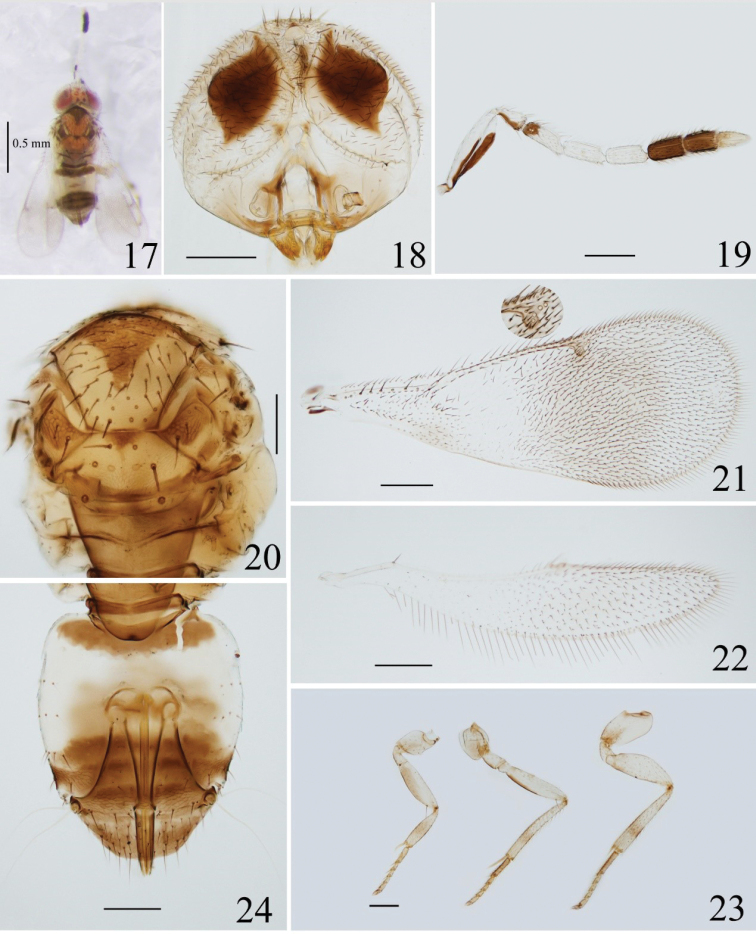
*Coccophagusperlucidus* sp. nov. **17** body, dorsal view **18** head **19** antenna **20** mesosoma **21** fore wing **22** hind wing **23** legs, from left to right: fore-, mid- and hind-leg **24** metasoma.

##### Diagnosis.

*Coccophagusperlucidus* sp. nov. can be distinguished from females of other species in this genus by the following combination of characters: scape (Fig. 19) slightly expanded, 3.1–3.3× as long as wide; fore wing infuscate posterior to stigmal vein, and with a hyaline area bearing sparse and transparent setae below basal half of marginal vein as in Fig. 21; the characteristic colour of mesosoma and metasoma as in Fig. 17.

##### Description.

**Female.** Length 0.9–1.4 mm; holotype, 1.4 mm.

***Colour*.** Head, in frontal view, mostly pale yellow, with mouth margin brown; in dorsal view, vertex orange, ocelli and eyes red, with two small dark patches behind each posterior ocellus (Fig. 17). Occiput suffused with brown above foramen, remainder pale yellow. Scape mostly white, and dark brown on ventral surface except distal one fourth white and having a pale streak on dark area as in Fig. 19; pedicel white except dark ventrally; funicle white except basal one third of F1 dark, C1 and C2 dark, C3 pale brown. Mandible brown. Pronotum largely dark brown except lateral sides yellow; mid lobe of mesoscutum (Figs 17, 20) with a large dark patch on anterior part, the remaining part orange; side lobe and axilla largely dark except lateral sides yellow; mesoscutellum orange except brown posteriorly, and with a small brown inverted triangle anteriorly; metanotum and propodeum brown and with lateral sides paler. Fore wing (Fig. 21) with stigma vein brown, largely infuscated below stigma vein, and with a hyaline area bearing sparse and transparent setae below basal half of marginal vein; hind wing (Fig. 22) slightly infuscate in distal half. Legs (Fig. 23) mostly yellow and with brown parts as following: procoxa apically, profemur ventrally, protibia medially, mesocoxa largely, ventral margin of mesofemur except distal one third, mesotibia submedially, metacoxa apically, metatibia submedially, all tarsomeres. Metasoma (Fig. 24) with petiole, Gt_1_ except posteriorly and Gt_5_–Gt_7_ dark brown, Gt_7_ pale brown anteriorly, Gt_2_ to Gt_4_ largely white except Gt_3_ having a short brown band medially. Ovipositor dark brown.

***Head*** (Fig. 18), in frontal view, 0.9× as high as wide. Ocellar triangle with apical angle acute. Mandible with two teeth and a truncation. Antenna (Fig. 19) with scape 3.1–3.3× as long as wide; pedicel 1.5–1.9× as long as wide, 0.7× length of F1; an anellus present between pedicel and F1; F1–F3 ventrally connected, F1 with ventral length a little longer than dorsal length, and 2.1–2.9× as long as wide; F2 slightly shorter than F1, 2.0–2.3× as long as wide; F3 nearly as long as F2, and 2.1× as long as wide; clava 0.8× length of funicle, and C1–C3 almost same in length, with C3 slightly narrower. Flagellomeres each with 2 longitudinal sensilla.

***Mesosoma*** (Fig. 20). Dorsum of mesosoma finely reticulate, and with the sculpture more evident in dark areas. Mid lobe of mesoscutum bearing approximately 70 setae, and with anterior setae short and dense, 0.8× as long as wide, 1.4× as long as mesoscutellum; each side lobe of the mesoscutum with 3 setae; each axilla with 3 or 4 setae; mesoscutellum 0.6× as long as wide, with 3 pairs of setae. Distance between anterior pair of scutellar setae 0.4× and 0.3× that between median and posterior pair respectively. Placoid sensilla mesad of the median scutellar setae, and the distance between placoid sensilla about equal to that of anterior scutellar setae. Metanotum as long as propodeum.

***Wings*.** Fore wing (Fig. 21) 2.5–2.6× as long as wide, marginal setae short. Costal cell 0.9–1.0× length of marginal vein, bearing 1 row of setae; submarginal vein with 8 long setae; marginal vein with 13 setae along anterior margin; postmarginal vein absent; stigmal vein (Fig. 21, inset) swollen posteriorly and with sensilla arranged in 2 lines. Hind wing (Fig. 22) 5.1–5.9× as long as wide, with marginal setae 0.4–0.5× wing width.

***Legs*** (Fig. 23). Mesotibial spur as long as corresponding basitarsus.

***Metasoma*** (Fig. 24). Lateral sides of Gt_5_, Gt_6_ and Gt_7_ clearly imbricate reticulated on dorsal surface. Setation of tergites on dorsal surface as followings: Gt_2_ with 3 setae on each side, Gt_3_ and Gt4 with 4 setae on each side respectively, Gt_5_ and Gt_6_ with 10 and 6 setae arranged in a line respectively, Gt_7_ with 18 setae nearly arranged in 3 lines. Gt_7_ 0.3× as long as wide. Ovipositor originating from base of Gt_3_, 1.2× as long as mesotibia, and not or slightly exerted. Second valvifer 1.6–1.8× as long as third valvula; the latter 1.4–1.5× as long as mesobasitarsus.

**Male.** Unknown.

##### Host.

Unknown.

##### Etymology.

The specific name refers to this species having a hyaline area on the fore wing.

##### Distribution.

China (Xishuangbanna of Yunnan Province).

##### Comments.

Although *C.perlucidus* sp. nov. is very similar to *C.equifuniculatus* in having similar antenna and thorax, the new species differs from the latter by two unambiguous characters: (1) fore wing of *C.perlucidus* obviously with a hyaline area bearing sparse and transparent setae below basal half of marginal vein (vs without the hyaline area, and with setae of disc dark, cf. fig. 91B in Huang 1994); (2) colour of gaster is different. We examined all materials belonging to the *Coccophagusvarius* group in hand, the colour of scape and gaster exhibit very little variation in conspecific individuals. *Coccophagusperlucidus* with Gt_1_ except posteriorly and Gt_5_–Gt_7_ dark brown, Gt_7_ pale brown anteriorly, Gt_2_ to Gt_4_ largely white except Gt_3_ having a short brown band medially as in Fig. 17 (vs Gt_1_ with a broad brown band medially, Gt_2_ and Gt_3_ with short brown band medially, Gt_4_–Gt_7_ mostly brown cf. fig. 91C in Huang 1994). This new species also resembles *C.lii* in having a similar colour of gaster and a hyaline area bearing fine setae below basal half of fore wing. It differs from the latter by the following combination of characters: (1) scape and pedicel with dark areas (vs completely white); (2) the dark patch on mid lobe of mesoscutum not touching the posterior margin of mesoscutum (vs touching cf. fig. 86A in Huang 1994); (3) mesoscutellum largely orange except brown anteriorly and posteriorly (vs with a large brown inverted T-shaped patch); (4) scape 3.1–3.3× as long as wide (vs 3.7×), pedicel 0.7× length of F1 (vs nearly as long as).

#### ﻿ Coccophagus yunnana

Taxon classificationAnimaliaHymenopteraAphelinidae

Wang, Huang & Polaszek

07FFCE85-018E-5FE2-9E64-8FDC908288B5

Figs 25–32

Coccophagus yunnana Wang, Huang & Polaszek, 2020: 1888. Holotype ♀, China, FAFU, not examined.

##### Material examined.

1♀ [on slide, C202009-1]; Yunnan Province; Xishuangbanna; Mengla County; Menglun Town; 21°54.28'N, 101°16.75'E; 629 m a.s.l.; 25 Apr. 2019; Z.-l. Bai, Z.-g. Chen, Y.-j. Lin, C. Wang, H. Yu leg.; LFNU. 2♀♀ [1♀ on slide, C202009-3; 1♀ destroyed for DNA extraction]; Yunnan Province; Xishuangbanna; Mengla County; Menglun Town; 21°54.33'N, 101°16.78'E; 616 m a.s.l.; 26 Apr. 2019; Z.-l. Bai, Z.-g. Chen, C. Wang, Y.-f. Tong, H. Yu leg.; LFNU. 2♀♀ [on slides, C201911-1, C201911-2]; Yunnan Province; Xishuangbanna; Mengla County; Menglun Town; 21°54.34'N, 101°16.79'E; 618 m a.s.l.; 2 May. 2019; Z.-l. Bai, Z.-g. Chen, C. Wang, Y.-f. Tong, H. Yu leg.; LFNU.

**Figures 25–32.**
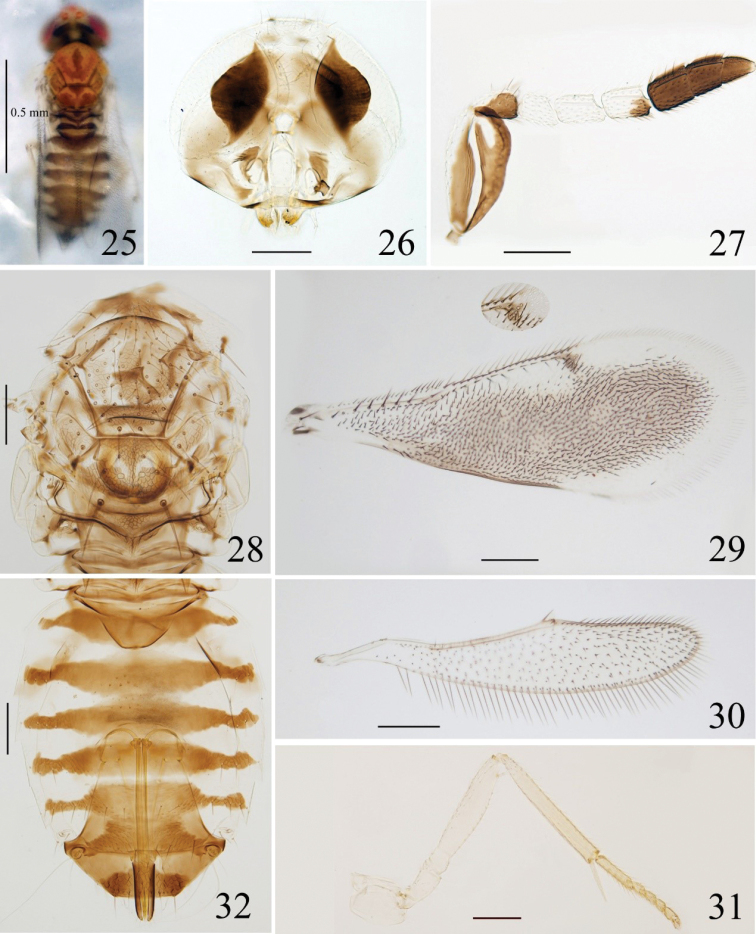
*Coccophagusyunnana***25** body, dorsal view **26** head **27** antenna **28** mesosoma **29** fore wing **30** hind wing **31** mid leg **32** metasoma.

Wang et al. (2020) provided abundant descriptions for this species based on a single female specimens reared from an unidentified coccid (Hemiptera, Coccidae) on *Kopsiafruticosa* (Ker). Here we provided some figures and DNA sequence for references.

##### Distribution.

China (Xishuangbanna of Yunnan Province).

## ﻿Phylogenetic analysis

**Figure 33.**
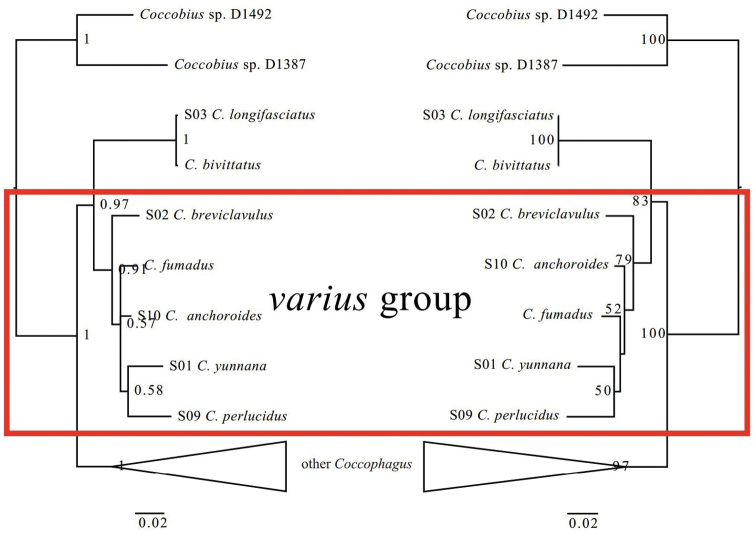
Phylogenetic trees constructed by Bayesian inference (BI) (left)/ Maximum likelihood (ML) (right) methods based on the 28S-D2 rDNA dataset. In the BI tree, all nodes of posterior probability (PP) value lower than 0.5 were shown as polytomy. In the ML three, all Branch support (BS) values lower than 50 were not shown. Detailed trees of both BI and ML were shown in Suppl. material 1: Figs S1, S2.

Phylogenetic relationship between *Coccophagusvarius* group and other *Coccophagus* species are shown in Fig. 33 and Suppl. material 1: Figs S1, S2. In this study, we provided 10 new 28S-D2 rDNA sequences of 10 species, representing 5 species groups as shown in Table 1. The *varius* group was found to be monophyletic in both BI and ML analysis with very strong support (PP=0.99; BS=91). Both resulting trees also lend support to the idea that *C.longifasciatus + C.bivittatus* (*ochraceus* group) serve as the sister group of *varius* group, which has been hinted at the analysis of Wang et al (2020). The 28S-D2 rDNA sequences between *C.longifasciatus* and *C.bivittatus* have no differences, these two species are very similar morphologically. They are maybe conspecific, which is first suspected by Hayat (1998). Our slide-mounted materials of *C.longifasciatus* agreed well with the original description. The material of *C.bivittatus* from Zhou et al. (2017) need to be checked in the future to verify if it is a misidentification of *C.longifasciatus*, and if not, then other gene regions (e.g. COI sequence) should be more indicative to test the possibility of *C.bivittatus* as a synonym of *C.longifasciatus. Coccophagusvarius* group and *C.ochraceus* group together form a monophyletic clade, being the sister group of the remaining *Coccophagus*. Though both resulting trees showed similar relationships between species groups, the relationships between species within groups were not fully resolved, which might be attributed to the conservative property of 28S rDNA. More species and genetic data of *Coccophagus* should be added to verify the monophyly of *varius* group and elucidate the relationships between *varius* group and other *Coccophagus* in the future.

## Supplementary Material

XML Treatment for Coccophagus anchoroides

XML Treatment for Coccophagus breviclavulus

XML Treatment for Coccophagus perlucidus

XML Treatment for Coccophagus yunnana

## Supplementary materials

Supplementary material 1 Figures S1, S2 Data type phylogenetic, images Explanation note Phylogenetic trees constructed by Bayesian inference (BI)/ Maximum likelihood (ML) methods based on the 28S-D2 rDNA dataset. https://binary.pensoft.net/file/666984This dataset is made available under the Open Database License (http://opendatacommons.org/licenses/odbl/1.0/). The Open Database License (ODbL) is a license agreement intended to allow users to freely share, modify, and use this Dataset while maintaining this same freedom for others, provided that the original source and author(s) are credited.Yao-guang Qin
