# Supplementary material for: ﻿On the genus Coccophagus Westwood (Hymenoptera, Aphelinidae) from Xishuangbanna Rainforest. Contribution I: Two new species of the Coccophagusvarius group, with an identification key and phylogenetic analysis
Source: Zookeys. 2022 Apr 1;1091:119–38. doi: 10.3897/zookeys.1091.80065 (PMC9005465; doi:10.3897/zookeys.1091.80065)
Supplement: Supplementary material 1 — Figures S1, S2 [file zookeys-1091-119-s001.pdf]

Supplementary Figures

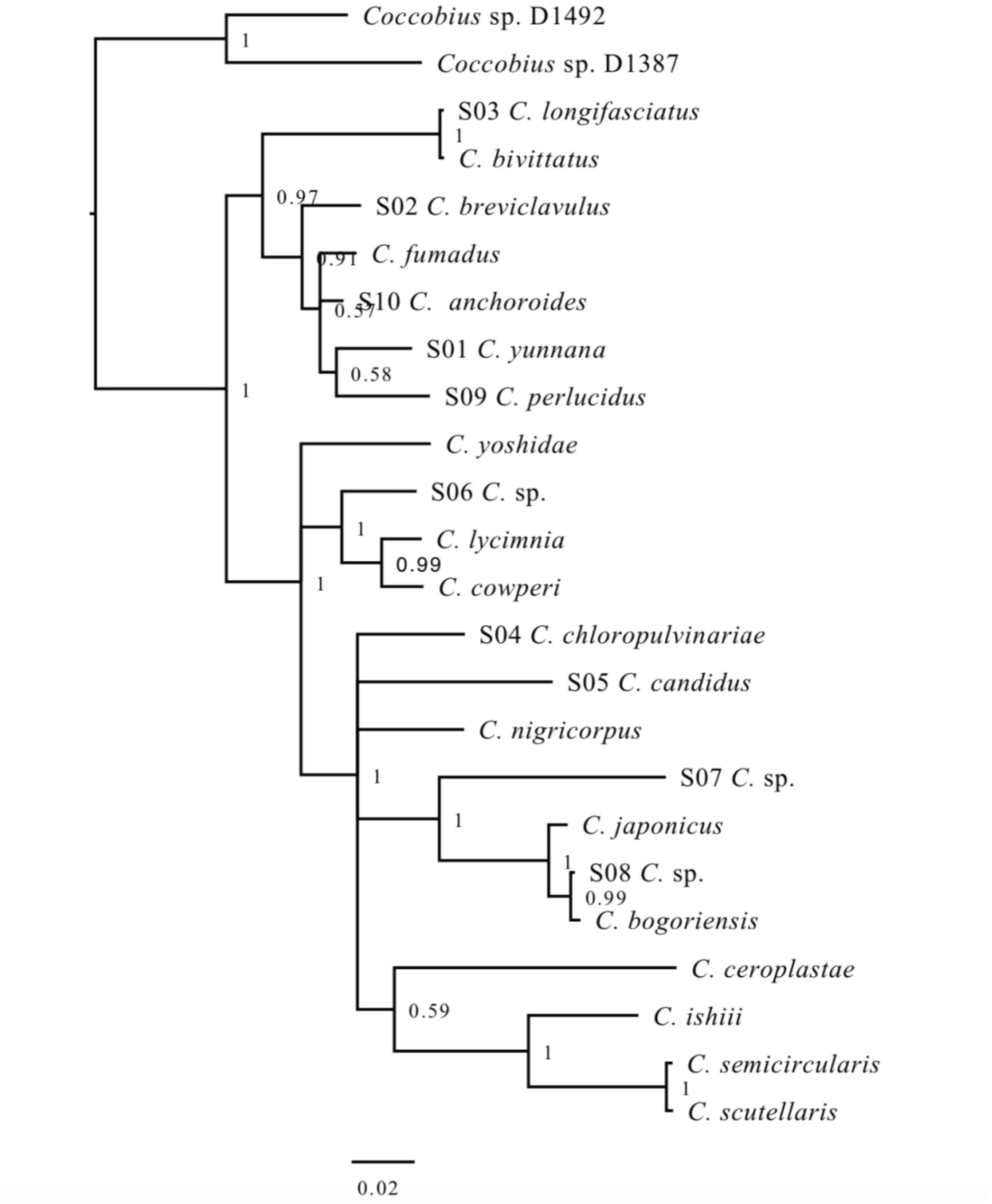

Supplementary Figure 1 Phylogenetic relationships among five species groups of genus *Coccophagus* by BI analysis based on the 28S-D2 rDNA dataset.

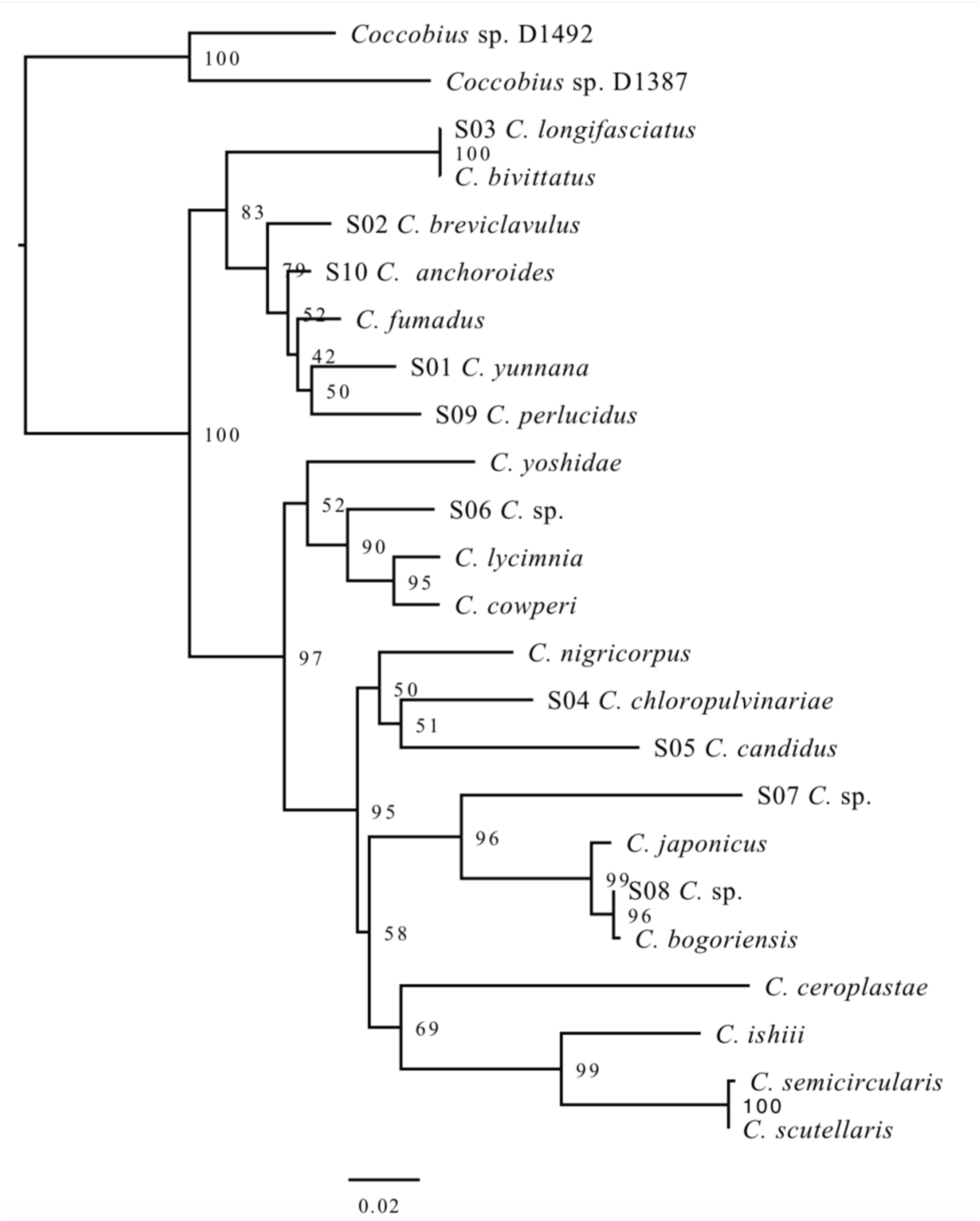

Supplementary Figure 2 Phylogenetic relationships among five species groups of genus *Coccophagus* by ML analysis based on the 28S-D2 rDNA dataset.
